# Pingmu Decoction Induces Orbital Preadipocytes Apoptosis In Vitro

**DOI:** 10.1155/2017/2109249

**Published:** 2017-04-18

**Authors:** Yali Zhang, Hong Li, Long Gao, Xia Zhang, RuiFang Xie

**Affiliations:** ^1^Department of Gastroenterology, Long Hua Hospital Affiliated to Shanghai University of Traditional Chinese Medicine, The Institute of Digestive Disease Affiliated to Shanghai University of Traditional Chinese Medicine, South Wan Ping Road No. 725, Shanghai 200032, China; ^2^Department of Endocrinology, Long Hua Hospital Affiliated to Shanghai University of Traditional Chinese Medicine, South Wan Ping Road No. 725, Shanghai 200032, China; ^3^Department of Pharmacy, Long Hua Hospital Affiliated to Shanghai University of Traditional Chinese Medicine, South Wan Ping Road No. 725, Shanghai 200032, China

## Abstract

Pingmu Decoction is the Traditional Chinese Medicine which has treated Graves' Ophthalmopathy (GO) in the inactive stage for more than ten years. This study was to explore the mechanism of Pingmu Decoction of inhibiting preadipocytes in GO patients from differentiating into mature adipocytes. Human orbital preadipocytes were isolated and cultured through tissue explant method. Orbital preadipocytes were induced into mature adipocytes. The medicinal serum was prepared from rats. The cells were treated with medicinal serum which were divided into three groups, low dose group (5%), medium dose group (10%), and high dose group (20%). The cells viabilities were observed by Oil Red O staining, MTT method, and Annexin V/propidium iodide (PI) double staining. Effect of Pingmu Decoction on cell apoptosis rate of orbital matured adipocytes was measured by flow cytometry. The genes Fas and Fas L from cell groups were tested by RT-PCR and Western blotting. The expression of master adipogenic transcription factors, including peroxisome proliferation-activity receptor (PPAR) *γ* and CCAAT/enhancer binding protein (C/EBP) *α*, was tested by Western blotting. Pingmu Decoction could reduce orbital preadipocytes viability and induce apoptosis of mature adipocyte via Fas/Fas L signaling pathway. Pingmu Decoction reduced lipid accumulation and downregulated the expression of PPAR *γ* and C/EBP *α*. Pingmu Decoction may play a therapeutic effect by reducing the accumulation of orbital adipocytes.

## 1. Introduction

Graves' Ophthalmopathy (GO), also known as thyroid-associated ophthalmopathy, is an autoimmune disease characterized by invasion of retrobulbar and orbital tissues [1, 2]. About 20–50% of GO patients also present clinically with Graves' Disease (GD), which is characterized by diffuse goiter with hyperthyroidism [3–5]. In recent years, the incidence of GD has undergone an annual increase due to the influence of hereditary and environmental factors that contribute to autoimmune disorders [6]. GO affects adults worldwide and has become one of the major causes of blindness. However, the etiology and pathogenesis of GO have not been completely defined. An increase in orbital adipose tissue content may increase the orbital pressure and lead to proptosis. Thus, abnormal proliferation of orbital adipose tissue and the occurrence of fat in GO play an important role in disease development [7, 8]. Furthermore, an increase in orbital preadipocyte volume and quantity may lead to the characteristic increase in adipose tissues [9]. Recent studies have confirmed that preadipocytes that reside in orbital tissues of patients can transform into fatty cells under certain conditions and contribute to the development of GO [10, 11]. Therefore, an imbalance in the proliferation, differentiation, and apoptosis of orbital preadipocytes may be the main factor regulating orbital exenteration increase, orbital pressure increase, and the occurrence of proptosis in patients with GO.

GO can be divided into active and inactive stages according to its development. In the inactive stage, the clinical curative effect is not very good, with no available specific and effective methods of treatment by Western medicine. Pingmu Decoction has been used in Chinese medicine to treat GO in the inactive stage for more than ten years, with good clinical curative effects. A preliminary experimental study demonstrated that serum from rats treated with Pingmu Decoction and its components can act on orbital preadipocytes from patients with GO in the inactive period. Pingmu Decoction serum promotes the apoptosis of adipocytes and reduces the accumulation of orbital adipocytes by increasing the expression of Caspase-3, Caspase-8, and Caspase-9 protein [12]. However, its apoptotic effect on differentiated adipocytes from GO patients remains uncharacterized. Intracellular pathways mediated by Fas/Fas L in mammals can induce apoptosis [13, 14]. We cultured orbital preadipocytes from patients by the tissue explant method and differentiated them into orbital adipocytes. The effect and molecular mechanism of Pingmu Decoction on the apoptosis of orbital adipocytes were explored by a variety of methods.

## 2. Materials and Methods

### 2.1. Experimental Animals and Drugs

Six-week-old specific pathogen-free male Sprague-Dawley rats weighing 200 ± 10 g were purchased from Shanghai Silaike Experimental Animal Co. Ltd. (certification number SCXK (Hu) 2012-0002). Animal experiment ethical approval was obtained from the Affiliated Long Hua Hospital of Shanghai University of Traditional Chinese Medicine.

Pingmu Decoction was composed of 30 g of Huangqi* (Astragalus membranaceus)*, 15 g of Xianlingpi* (Herba Epimedii)*, 15 g of Chuanxiong* (Ligusticum wallichii)*, 15 g of Baijiezi* (Brassica alba Boiss.)*, 15 g of stir-baked Biejia (Carapax Trionycis), and 30 g of Cheqianzi* (Semen plantaginis)*. These herbs were provided by the Chinese Medicine Pharmacy of the Affiliated Long Hua Hospital of Shanghai University of Traditional Chinese Medicine (TCM). The Decoction of the above herbs was prepared twice according to the original formula and then filtered and concentrated in a combined liquor. The dose of rat administration is based on the human mouse equivalent dosage conversion according to the textbook* Pharmacology and Pharmacology Experiment of TCM* (edited by Zhang Dafang, Shanghai Science and Technology Press, 2002). The crude drug (4 g/mL) was stored at 4°C.

### 2.2. GO Patients

Four cases (4 eyes) of postbulbar adipose tissues from GO inpatients were collected from Shanghai EENT Hospital. Of the four GO cases in this study, two female and two male patients (age ranging from 25 to 48 years old) had a GO history of three to eight years. Diagnostic criteria of GO were in accordance with the clinical score standards described in the* Chinese Guideline for Diagnosis and Treatment of Thyroid Disease *published in 2008. The clinical activity score (CAS) of GO in clinical practice was based on the clinical score standards of* Chinese Guideline for Diagnosis and Treatment of Thyroid Disease*, including spontaneous retrobulbar pain, pain with eye movement, eyelid erythema, conjunctival hyperemia, chemosis, swollen lacrimal caruncle, and swollen eyelid. Each symptom accounted for one point, and the total score obtained was up to seven in each case. The inactive phase of GO referred to active exophthalmos with the clinical score of <3. Classification of exophthalmos in this study was in accordance with the classification criteria of GO proposed by the American Thyroid Association (ATA). The patients were confirmed to have GO paralleled orbital decompression by the Ophthalmology Department at Shanghai EENT Hospital. All patients were in the GO inactive stage, which is in line with the diagnostic criteria. Other diseases, such as autoimmune diseases and cancer, were ruled out. Approval was obtained from the Medical Ethical Committee and patient consent was obtained in writing prior to collection of tissue samples.

### 2.3. Medicinal Serum Preparation

The Sprague-Dawley rats were randomly divided into 2 groups with 5 rats per group. The rats in the Pingmu Decoction group were given 15 mL/kg Pingmu Decoction, and the rats in the control group were given an equal volume of distilled water by intragastric administration twice a day for 3 days. The final concentrations of medicinal serum (final concentration = volume added to serum/total volume × 100%) were 5% (low dose), 10% (medium dose), and 20% (high dose).

Blood was collected from the abdominal aortae of the rats under ether anesthesia 1.5 h after the last administration. The blood was incubated for 2 h at 4°C and then centrifuged at 4°C at 3000 rpm/min for 15 min. The sera from each group of mice were combined and inactivated for 30 min at 56°C in a water bath, filtered with 0.22 *μ*m aperture micropore film, packed with frozen pipes, and stored at −20°C.

### 2.4. Culture of Primary Cells from Patient Tissue

Sterilized orbital adipose tissues from the operating table were washed 3 times with sterile phosphate buffer solution (PBS) to remove the blood and then were placed into culture bottles containing complete medium. As soon as possible after transfer to the laboratory (within 4 h) the tissues were processed for experimentation at room temperature. Human orbital preadipocytes were cultured by the tissue explant method. The adipose tissue with visible connective tissue or blood clots was removed and the remaining tissue was harvested into Petri dishes with D-Hank's solution. The tissues were cut into pieces of approximately 1 mm × 1 mm × 1 mm in size with ophthalmic scissors and were seeded evenly at the bottom of culture bottles. Then the bottles were gently turned upside down, and 2 mL complete culture solution was added. The bottles were placed askew into an incubator with 5% CO_2_ at 37°C for culturing. After the tissue explants adhered tightly to the culture bottle, 5 mL complete culture solution was added, and the solution was replaced with fresh solution every 2-3 d. At 80% confluence, the cells were passaged 1 : 3 (every 3-4 d). Cells were used in experiments after 3–6 generations. The morphology and growth status of the cultured primary cells were observed every day. Oil Red O staining and microscopic photography were performed to assess the formation of lipid droplets. Healthy preadipocytes were seeded at a density of 3–5 × 10^7^/L on coverslips in a 6-well plate. When they approached confluence, the cells were fixed in 4% paraformaldehyde for 10 minutes at normal temperature and stained by indirect immunofluorescent assay. The prepared cell slides were washed 3 times with PBS for 3 minutes, incubated for 1 h at 37°C with PBS containing 2% bovine serum albumin and Pref-1 antibody (diluted 1 : 100), and then incubated at 4°C overnight. After 4 washes with PBS for 5 minutes, FITC-secondary antibody (diluted 1 : 500) was added for 30 min at room temperature in darkness and the slides were washed 4 times with PBS for 5 minutes. The slides were then stained with 5 *μ*g/mL DAPI for 2 min, and antiquenching mounting medium was added before examination under a fluorescent inverted microscope.

### 2.5. Identification of Mature Adipocytes

Healthy preadipocytes were seeded at a density of 1 × 10^5^ in 6-well plates. After the cells adhered, the medium was replaced with Differentiation Medium I containing Dexamethasone (1 *μ*mol/L), IBMX (0.2 mmol/L) and insulin (10 *μ*g/mL) to induce differentiation. After 4 days, the medium was replaced with Differentiation Medium II, which has no Dexamethasone and IBMX, and the cells were cultured for 4 additional days. Then medium was replaced with complete medium and the cells were cultured for 8 d, with the medium replaced every 2 d. The entire differentiation period was 16 d.

After differentiation, the mature adipocytes were washed 3 times with PBS and fixed with 4% paraformaldehyde for 10 minutes. The excess liquid was removed by aspiration, and Oil Red O solution was added. The cells were rinsed with 75% ethanol to facilitate observation. The resulting orange lipid droplets were observable for 10 min under the fluorescent inverted microscope.

### 2.6. Cell Viability Assessment by the MTT Method

Cells in the logarithmic phase were digested with trypsin to produce a monocell suspension in complete culture medium. The cells were seeded in 96-well plates at about 10000 cells/200 *μ*L per well. Then the 96-well plates were cultured in 5% CO_2_ in an incubator at 37°C. The cells in each group (group 1: control group, group 2: Pingmu Decoction low dose group, group 3: Pingmu Decoction medium dose group, group 4: Pingmu Decoction high dose group, and group 5: Dexamethasone group) were cultured for 24 h, 48 h, 72 h, and 96 h. A zero control (culture media, MTT, and DMSO) and a blank control (cells, culture medium without serum, MTT, and DMSO) were also assayed in triplicate. After 4 additional days of culture, 20 *μ*L MTT solution (5 mg/mL in PBS) was added to each well. The culture supernatant was carefully aspirated, and then 150 *μ*L DMSO was added to each well with low speed oscillation to allow the crystals to dissolve adequately. The optical density value of each group was measured at 490 nm, and a cell growth curve was drawn using time as the *x*-axis and light absorption as the *y*-axis.

### 2.7. Apoptosis Assessment by the Annexin V/Propidium Iodide (PI) Double Staining Method

Cells treated with different amounts of Pingmu Decoction serum were cultured for 2 d. The cell culture medium in each well of a 6-well plate was aspirated, and 400 *μ*L trypsin was added. After 3 min, 1 mL complete culture medium was added to terminate the digestion and the nonadherent cells were collected. The wells were washed with 500 *μ*L culture media to recover all of the cells, and cells from both washes were combined and centrifuged at 2000 rpm/min for 5 min. The supernatant was discarded, and the cells were washed with chilled sterile PBS, resuspended in PBS, counted, and adjusted to 1 × 10^6^/mL. The cell suspensions were collected, mixed gently with 5 *μ*L Annexin V-FITC, and incubated at room temperature for 15 min in darkness. Then 5 *μ*L PI staining solution was added gently, and the cells were incubated in an ice bath in darkness for 5 min. Flow cytometry was performed immediately after adding 300 *μ*L binding buffer.

### 2.8. Real-Time PCR Assessment of Fas and Fas L mRNA Expression

Total mRNA was extracted by the Trizol method. The samples (2 *μ*L per well) were plated, with DEPC water as the background. Then the sample concentration was assessed using a micro reader. RNA of sufficient purity (A260/A280 value 1.8–2) was used for subsequent experimentation. The concentrations were adjusted to 1 *μ*g/*μ*L, and then mRNA was heat-denatured for 5 min at 65°C, cooled immediately, and reverse transcribed to cDNA using the Sensiscript RT Kit. Reverse transcription was performed in a 20 *μ*L volume (9 *μ*L nuclease-free water, 4 *μ*L 5x RT Buffer, 1 *μ*L RT Enzyme, 1 *μ*L Primer Mix, and 5 *μ*L cDNA). Reaction conditions were as follows: 37°C for 15 min, 98°C for 5 min, and 4°C for 5 min.

For PCR, the primer sequences selected were designed based on the information in GenBank. The upstream and downstream sequences of the primers and predicted product size were as follows: Fas: 5′-TTCTGCCATAAGCCCTGTCC-3′ and 5′-CTAAGCCATGTCCTTCATCACAC-3′ (169 bp); Fas L: 5′-GGATGTTTCAGCTCTTCCACCTAC-3′ and 5′-TGTTAAATGGGCCACTTTCCTC-3 (152 bp); GAPDH: 5′-GCACCGTCAAGGCTGAGAAC-3′ and 5′-TGGTGAAGACGCCAGTGGA-3′ (138 bp). Real-time PCR reactions (50 *μ*L total) included 5 *μ*L cDNA sample, 2 *μ*L upstream primers (10 *μ*M), 2 *μ*L downstream primers (10 *μ*M), 16 *μ*L distilled water, and 25 *μ*L SYBR Green. The conditions of PCR amplification were 95°C for 15 s, 60°C for 15 s, and 72°C for 45 s (40 cycles). The relative quantity of the target genes was determined by the comparative Ct value/2^−ΔΔCt^ method with GAPDH as control.

### 2.9. Assessment of Fas, Fas L, PPAR *γ*, and C/EBP *α* Protein Expression by Western Blotting

Cells were cultured for 2 d under different conditions (control group, Pingmu Decoction low, moderate, and high dose group, and Dexamethasone group). 2 × 10^6^ adipocytes were washed twice with PBS, and 100 *μ*L precooling protein lysis buffer (50 mM Tris-HCl, 150 mM NaCl, 0.02% NaN_3_, 1% Triton X-100, 1 mM PMSF, 1 *μ*g/mL aprotinin, and 1 *μ*g/mL leupeptin) was added to the plates on ice for 10–20 min. The cells were then scraped and collected into EP tubes at 12000 rpm for 5 min at 4°C. Then the supernatants were transferred into another centrifuge tube, and a small amount of supernatant was used for protein quantitation by the Bradford Assay. Fifty microliters of protein from each sample was separated by SDS-PAGE, and after transfer, PVDF membranes were incubated at 4°C overnight with primary antibody against Fas, Fas L, PPAR *γ*, or C/EBP *α* (1 : 1000; diluted in TBST). Fluorescence-labeled secondary antibody (1 : 2000) was added after washing, and the membranes were incubated for 2 h at room temperature. The membranes were rinsed with TBST. ECL luminescence reagents (reagent A mix with equal volume of reagent B) were added, and the membranes were dynamically integrated on a gel imaging instrument for 5–15 min with analysis by Gel-Pro analysis software.

### 2.10. Statistical Analysis

Statistical analysis was performed using SPSS 18.0 software. Data measurement was expressed as mean values ± standard deviation. Normal distribution and homogeneity of variance assessments were carried out first. For experimental data that met the criteria, comparisons between multiple groups were performed using single-factor analysis of variance (analysis of variance, ANOVA); and comparisons among groups were performed using LSD-*t* (least significant difference-*t*). For experimental data that did not meet the normal distribution and homogeneity of variance criteria, nonparametric tests were adopted. *P* < 0.05 was considered statistically significant.

## 3. Results

### 3.1. Primary Culture of Preadipocytes, Growth Characteristics after Passage, and Morphological Observation

To determine the molecular effects of Pingmu Decoction on orbital adipocytes from GO patients, we collected tissue from patients undergoing surgery. Cells cultured from the tissue samples emerged from the tissue block on the fourth day and were spindle shaped with oval or near-circular nuclei. The cells morphology was close to that of fibrocytes, with gradual proliferation of cells. After passaging, the preadipocytes gradually arranged themselves and grew 3-4 d before requiring subsequent passage. In this study, orbital adipose tissue from 4 cases of GO was cultured, and cells emerged from the adipose tissue block in all cases within 4-5 d. The cells were passaged when they reached confluence (within 9-10 d) under the sterile conditions. Subsequently, the cells were passaged once every 3-4 d.

### 3.2. Identification of Preadipocytes

**Figure 1:**
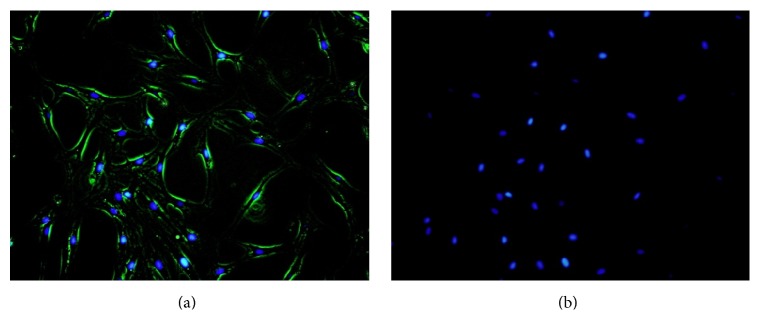
Verification of Pref-1 expression by preadipocytes. Positive Pref-1 expression by preadipocytes was confirmed by indirect immunofluorescent assay (a-b). Merged image of Pref-1 stain (green) and nuclear stain (blue) (a). Nucleus was stained by DAPI (blue) (b).

To verify that the cultured cells represented preadipocytes, we performed immunofluorescent staining assays with Pref-1 antibody. The membranes of the cultured preadipocytes positively expressed Pref-1 (green fluorescence) as identified by indirect immunofluorescent assay (Figures 1(a) and 1(b)). Based on this finding, it can be verified that the cultured primary cells were preadipocytes.

### 3.3. Induction of Differentiation of Preadipocytes and the Effects of Pingmu Decoction on Differentiation

**Figure 2:**
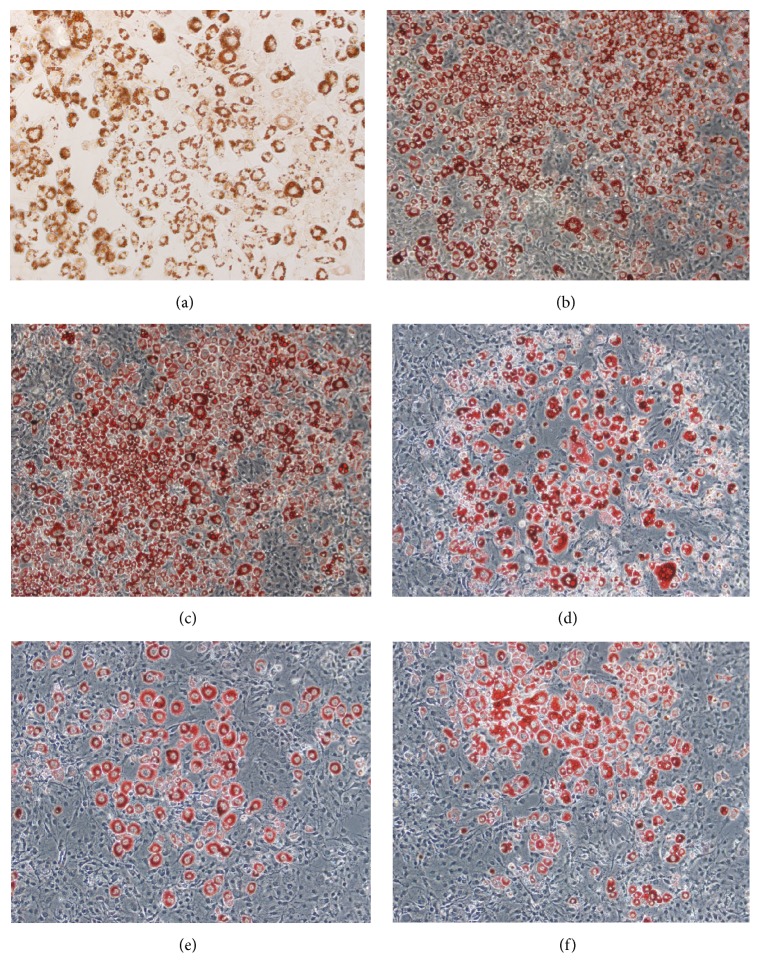
Fat accumulation in mature adipocytes. Lipid droplets in adipocytes were stained with Oil Red O. Staining was performed for normal mature adipocytes (a) and for preadipocytes treated with or without Pingmu Decoction serum during differentiation as indicated (b–f). Blank, blank control group; Dexa, Dexamethasone group; Pingmu low, Pingmu Decoction low dose group; Pingmu moderate, Pingmu Decoction medium dose group; Pingmu high, Pingmu Decoction high dose group.

To induce differentiation of the preadipocytes into mature adipocytes, we cultured them in differentiation liquid. The cells retracted, the cell space became larger, and proliferation stopped gradually. Furthermore, the morphology of the cells gradually changed to oval or nearly circular, and the volume of the cells gradually increased. An obvious change in morphology was generally observed after a week or so. Lipid granules presented as fat droplets in the cytoplasm as observed under the light microscope. On the 8th day of the differentiation, the cells swelled prominently and their shape became short or polygonal. Fat droplets became enlarged and gradually increased in number as observed by Oil Red O staining. On the 16th day of the differentiation, the cell differentiation was close to 80–90% complete, and the nuclei shifted to one side. Fat droplets in the cytoplasm stained red with Oil Red O and could be seen under the inverted microscope (Figure 2(a)). Based on this finding, it could be verified that the preadipocytes were successfully differentiated as mature adipocytes.

Next, we treated the mature adipocytes with three different doses of Pingmu Decoction serum (low, 5%; medium, 10%; and high, 20%). As a control, cells were untreated (blank) or treated with 30 *μ*g/mL Dexamethasone (Dexa). The results demonstrate that Pingmu Decoction serum can inhibit differentiation (Figures 2(b)–2(f)). These findings suggest that the explanted preadipocytes can be differentiated, but that exposure with Pingmu Decoction serum during the differentiation blocks maturation.

### 3.4. Effect of Pingmu Decoction Serum on Cell Viability

**Figure 3:**
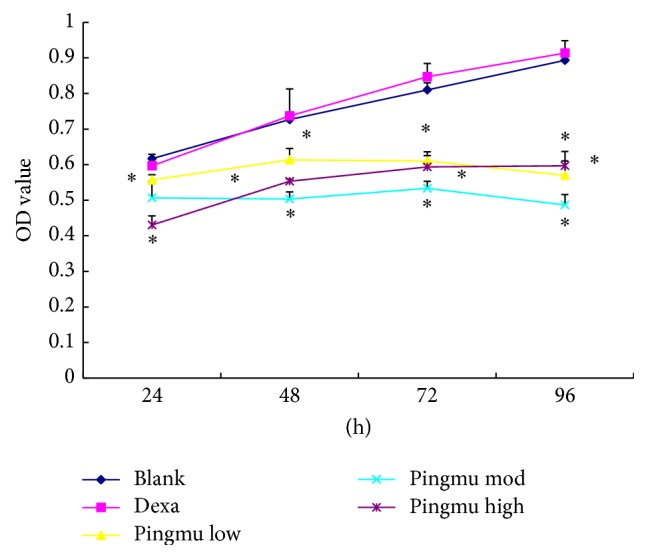
MTT cell viability assay of control adipocytes and adipocytes treated with Pingmu Decoction serum. Blank, blank control group; Dexa, Dexamethasone group; Pingmu low, Pingmu Decoction low dose group; Pingmu moderate, Pingmu Decoction medium dose group; Pingmu high, Pingmu Decoction high dose group. ^*∗*^*P* < 0.05 versus the blank control group (*n* = 3 per group).

To further explore the effects of Pingmu Decoction serum, we treated differentiated adipocytes for up to 96 h. The growth curves of the Pingmu Decoction low, moderate, and high groups, compared with the Blank and Dexa groups, were significantly reduced, which indicates that the cell viability was decreased (Figure 3). There were no significant differences between the Blank and Dexa groups. The OD values of each Pingmu Decoction group compared with the control group were decreased at 48 h (*P* < 0.05). The OD values were further decreased in the Pingmu Decoction groups at 96 h, with the most dramatic decrease observed for the medium dose group (*P* < 0.05). These results verify the time-dependent effect of Pingmu Decoction serum in suppressing the growth of adipocytes from GO patients.

### 3.5. Effect of Pingmu Decoction Serum on Apoptosis of Mature Adipocytes

**Figure 4:**
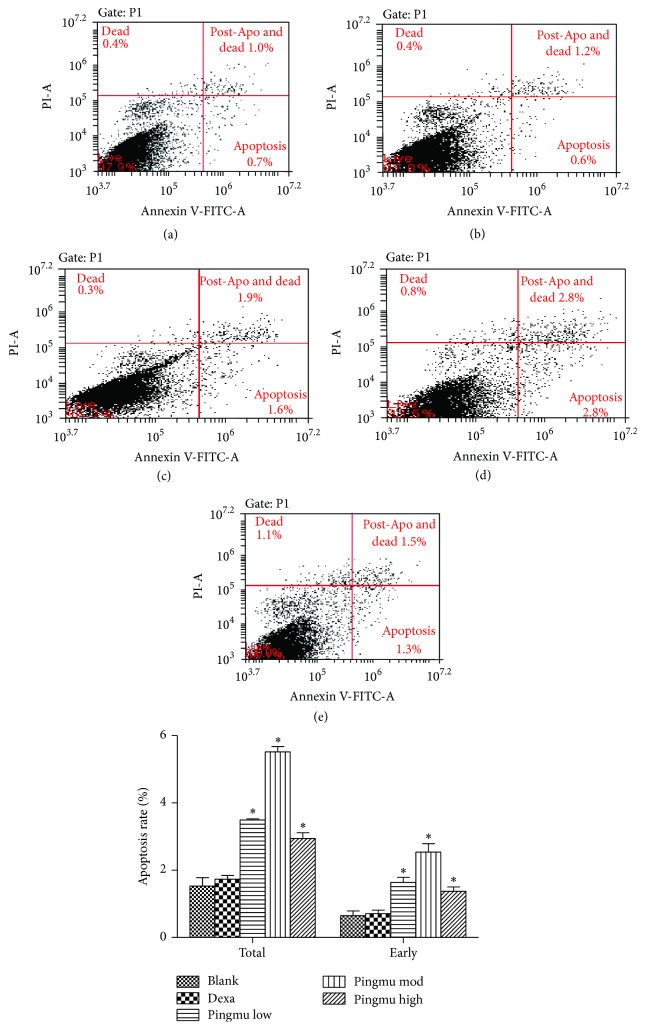
Apoptosis rates of the matured adipocytes treated with Pingmu Decoction serum. Apoptosis rates were determined by Annexin V/PI staining and flow cytometry. Blank control group (a); Dexamethasone group (b); Pingmu Decoction low dose group (c), Pingmu Decoction medium dose group (d); Pingmu Decoction high dose group (e). ^*∗*^*P* < 0.05 versus the blank control group (*n* = 3 per group).

To determine whether the decrease in cell viability by Pingmu Decoction serum occurs at the level of apoptosis, we performed flow cytometry of cells assessed by Annexin V/PI double staining. The results show that the early and total apoptosis rates of mature adipocytes were 0.65% and 1.53% in the blank control group. However, the cell apoptosis of each Pingmu Decoction group was increased (*P* < 0.05), with the early and total apoptosis rates in the Pingmu Decoction medium dose group being the highest (Figure 4). There were no significant differences between Dexa and blank groups. There were statistical differences between the medium dose group and low dose group (*P* < 0.05), but not the high dose group, in the early apoptosis rate. These results suggest that Pingmu Decoction serum induces apoptosis of mature GO adipocytes, with the most dramatic effect occurring at the medium dose.

### 3.6. Effect of Pingmu Decoction Serum on the Expression of Fas and Fas L mRNA

**Figure 5:**
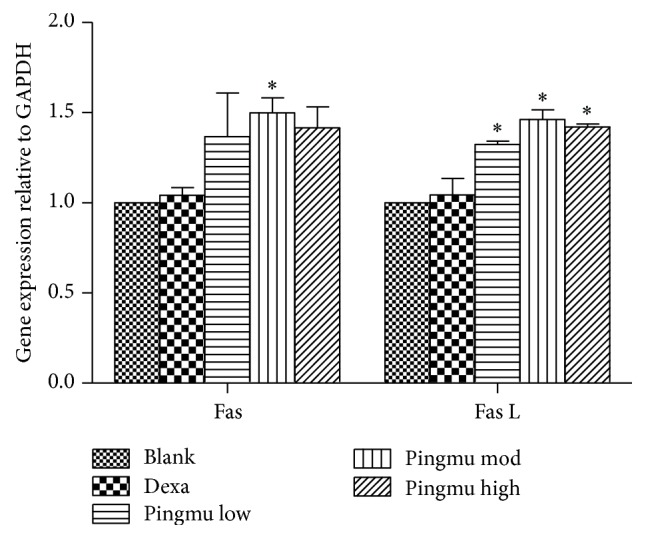
Effect of Pingmu Decoction serum on the expression of Fas and Fas L mRNA. Expression was quantified by RT-PCR. Blank, blank control group; Dexa, Dexamethasone group; Pingmu low, Pingmu Decoction low dose group; Pingmu moderate, Pingmu Decoction medium dose group; Pingmu high, Pingmu Decoction high dose group. ^*∗*^*P* < 0.05 versus the blank control group (*n* = 3 per group).

To further verify the apoptotic effect of Pingmu Decoction serum and to determine whether the effect might be explained by alterations in the expression of the apoptosis modulators Fas and Fas L, we performed RT-PCR. The relative expression of Fas mRNA in adipocytes from each Pingmu Decoction group was higher than blank control group (low: 1.37-fold increase; medium: 1.50-fold increase; high: 1.42-fold increase), and the difference between the low dose group and the blank group was statistical (*P* < 0.05). Furthermore, the relative expression quantity of Fas L mRNA in adipocytes from each Pingmu Decoction was higher than blank control (*P* < 0.05) (low: 1.32-fold increase; medium: 1.46-fold increase; high: 1.42-fold increase) (Figure 5). There were no significant differences in the expression of Fas or Fas L mRNA between the Blank and Dexa groups.

### 3.7. Effect of Pingmu Decoction Serum on the Expression of Fas and Fas L Protein

**Figure 6:**
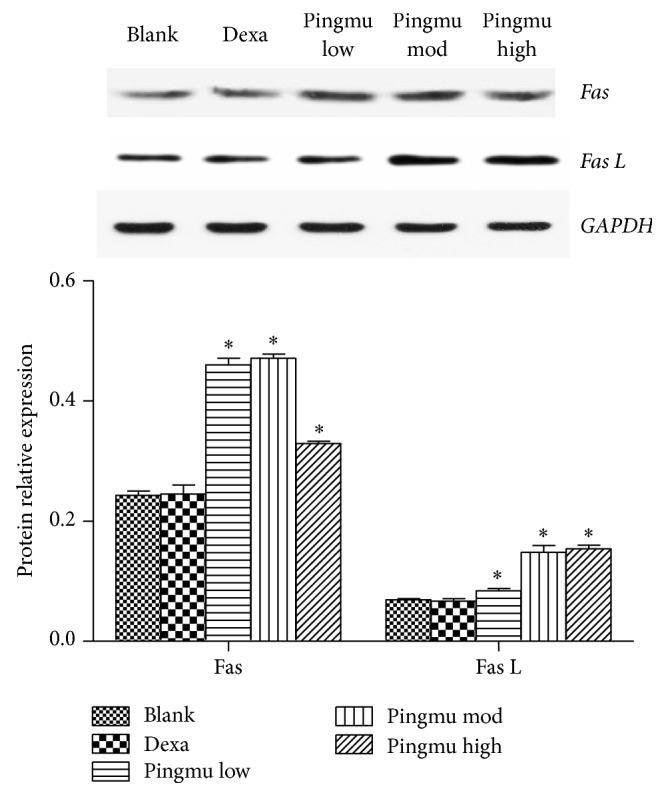
Effect of Pingmu Decoction serum on the expression of Fas and Fas L protein. Protein expression was quantified by Western blotting. Blank, blank control group; Dexa, Dexamethasone group; Pingmu low, Pingmu Decoction low dose group; Pingmu moderate, Pingmu Decoction medium dose group; Pingmu high, Pingmu Decoction high dose group. ^*∗*^*P* < 0.05 versus the blank control group (*n* = 3 per group).

The protein expression of Fas and Fas L was further assessed by Western blotting. Compared with the blank control group, the Fas and Fas L expression of each Pingmu Decoction group was increased statistically (*P* < 0.05) (Figure 6). Among the Pingmu Decoction groups, the low dose group had the highest expression of Fas but the lowest expression of Fas L, and the high dose group has the lowest expression of Fas but the highest expression of Fas L. There was no significant difference between the Blank and Dexa groups. These results verify the RT-PCR results suggesting that Pingmu Decoction may induce apoptosis of GO adipocytes by increasing Fas/Fas L signaling but suggest that the relationship may not be strictly dose-dependent.

### 3.8. Pingmu Decoction Decreases Protein Expression of Adipogenic Transcription Factors

**Figure 7:**
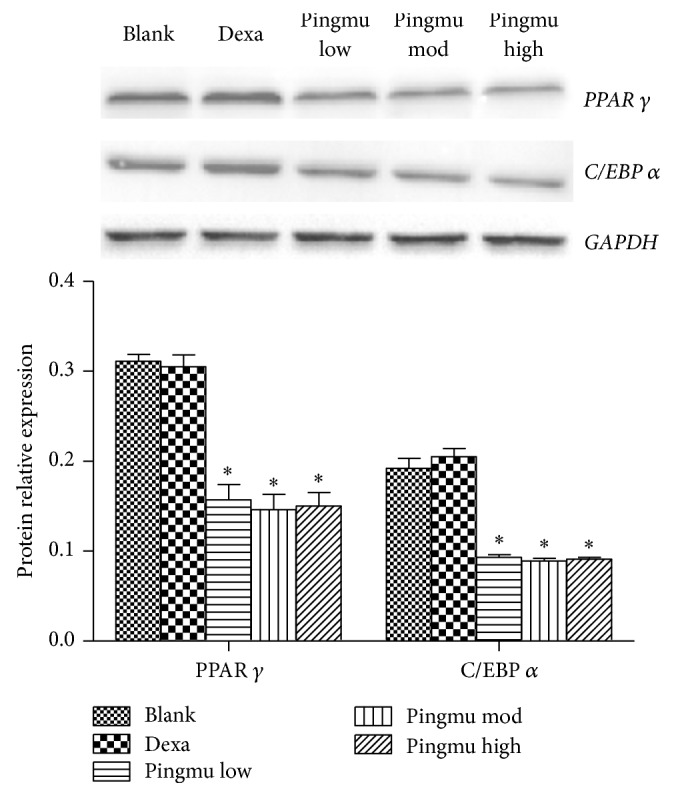
Effect of Pingmu Decoction serum on the expression of PPAR *γ* and C/EBP *α* protein. Protein expression was quantified by Western blotting. Blank, blank control group; Dexa, Dexamethasone group; Pingmu low, Pingmu Decoction low dose group; Pingmu moderate, Pingmu Decoction medium dose group; Pingmu high, Pingmu Decoction high dose group. ^*∗*^*P* < 0.05 versus the blank control group (*n* = 3 per group).

To identify the effect of Pingmu Decoction on adipogenic transcription factors, the protein expression of PPAR *γ* and C/EBP *α* was further assessed by Western blotting. Compared with the blank control group, the PPAR *γ* and C/EBP *α* expression of each Pingmu Decoction group was decreased statistically (*P* < 0.05) (Figure 7). Among the Pingmu Decoction groups, there was no significant difference. And also there was no significant difference between the Blank and Dexa groups.

## 4. Discussion

The clinical symptoms of patients with GO are caused by a shift in the balance between limited orbital volume and increasing amounts of the orbital adipose tissue [15]. The increase in orbital adipose tissue can directly increase orbital pressure. The abnormal proliferation of orbital adipose tissue and fat accumulation play a major role in the occurrence and development of GO. Accordingly, the role of orbital adipose tissue in GO has received increasing attention [16–18]. CT examination has indicated that the increase in orbital fat in GO is consistent with the degree of exophthalmos and inconsistent with orbital muscle enlargement [19]. Potgieser and others [20] have demonstrated that the increase in extraocular muscle volume in GO is obviously lower than the increase in orbital adipose tissue volume, and the correlation coefficient between total orbital adipose volume and the degree of exophthalmos is higher than the correlation coefficient between extraocular muscle volume and exophthalmos. Kumar and others [21] found evidence of new adipose tissue in GO patients. Furthermore, some GO patients with obvious exophthalmos are observed to only manifest with an increase in the retrobulbar adipose tissue, without the enlargement of extraocular muscle. Patients with this variety of GO mainly manifest with exophthalmos, which will affect appearance. In this case, the patients' total cross-sectional area of extraocular muscle is not large [22, 23].

To examine the mechanisms of growth control in adipocytes by Pingmu Decoction serum, we cultured preadipocytes. Preadipocytes were shown to be cultured successfully by the tissue explant method in this study. Cells emerged from the adipose tissue block on the 4th day. The primary cells were spindle shaped with oval or near-circular nuclei, which is similar to the morphology of fibroblasts under the microscope. The cells proliferated gradually at first and then proliferated more rapidly after passage. We verified the identification of human preadipocytes from the primary culture of orbital adipose tissue by staining for Pref-1, a marker expressed preferentially in preadipocytes, rather than mature adipocytes. Pref-1 inhibits the differentiation of the preadipocytes and reduces the generation of fat [24], and glucocorticoid can promote the differentiation of the adipocytes by inhibiting the expression of Pref-1 [25]. Our examination revealed that Pref-1 was positively expressed in cultured cells, which verifies that these cells were preadipocytes. Oil Red O staining, a specific method for staining lipid droplets, also was used to confirm that preadipocytes could be differentiated into mature adipocytes, which further validates our isolation and culture methods.

The effects of Pingmu Decoction on cell viability were assessed by MTT assay. Our results show that the cell viability of preadipocytes could be reduced by Pingmu Decoction serum at low medium and high doses at 48 h, with the most significant effects observed with the medium dose and at 96 h. The results of flow cytometry revealed that the early and total apoptosis rates of matured adipocytes were also increased obviously for cells exposed to Pingmu Decoction serum (*P* < 0.05), with the most dramatic effects in the medium and low dose groups. These results suggest that Pingmu Decoction can promote the apoptosis of adipocytes from GO patients. In these experiments, the medium dose, which was designed to reflect the prescribed human dose, was most effective. However, additional experiment with a more narrow range of doses might verify a dose-effect relationship.

We also demonstrated that Pingmu Decoction serum mediates an increase in Fas and Fas L, which was shown both at the level of mRNA and protein. Thus, the mechanism by which Pingmu Decoction serum may act to promote cell death could involve the activation of death receptor apoptosis mediated by Fas and Fas L expression. While the current study was limited in the availability of primary tissues, future experimentation should be performed to investigate the responsiveness of the primary adipocytes to Fas L, as well as the Fas L concentration in the orbital locale of patients as compared to healthy subjects. Pingmu Decoction reduced lipid accumulation and downregulated the expression of PPAR *γ* and C/EBP *α*. Icariin is the main constituent of Herba Epimedii. Han et al. found that Icariin can inhibit the adipocyte differentiation through downregulation of the adipogenic transcription factors [26]. Zhang et al. show that Icariin could inhibit adipogenic differentiation of BMSCs in the osteoblast-osteoclast coculture [27]. And Icariin can also inhibit the adipogenic transdifferentiation of osteoblasts [28]. However, despite experimental limitations, our study is consistent with the possibility that Pingmu Decoction reduces the accumulation of orbital adipocytes in the inactive stage of GO to play a therapeutic role, and this effect is likely to be explained by increased Fas/Fas L expression and increased apoptosis.

In this study, we used standardized methods to produce the Pingmu Decoction serum. Components used to make the Pingmu Decoction were produced in factories that adhere to TCM good manufacturing practice. Additionally, our protocols followed methods that are used in clinical application as described in the* Pharmacology and Pharmacological Experiments with Traditional Chinese Medicine* [29]. However, as for any form of herbal medicine, batches of Pingmu Decoction may vary according to the source. For this reason, future studies should be done to verify the findings using Pingmu Decoction from other sources.

## 5. Conclusion

Our results show that Pingmu Decoction can reduce the cell viability of orbital preadipocytes and inhibit their differentiation into adipocytes. This effect may be mediated by the activation of Fas and Fas L by the death signaling pathway to promote the apoptosis of matured adipocytes. These results suggest a therapeutic mechanism for Pingmu Decoction in reducing the accumulation of orbital adipocytes in GO.
